# Magnetic Resonance Imaging Signal Alterations in Paraspinal Muscles in Dogs with Acute Thoracolumbar Intervertebral Disk Extrusion

**DOI:** 10.3389/fvets.2018.00016

**Published:** 2018-02-15

**Authors:** Peter Trampus, Christine Goepfert, Monika Welle, Diana Henke, Franck Forterre, Daniela Schweizer-Gorgas

**Affiliations:** ^1^Division of Clinical Radiology, Vetsuisse-Faculty, University of Bern, Bern, Switzerland; ^2^Institute of Animal Pathology, Vetsuisse-Faculty, University of Bern, Bern, Switzerland; ^3^Division of Clinical Neurology, Vetsuisse-Faculty, University of Bern, Bern, Switzerland; ^4^Division of Small Animal Surgery, Vetsuisse-Faculty, University of Bern, Bern, Switzerland

**Keywords:** magnetic resonance imaging, paraspinal muscle, thoracolumbar, intervertebral disk extrusion, dog, signal alteration

## Abstract

Muscle signal alteration detected on MRI is seen in diverse pathologic conditions. We observed signal alterations within the paraspinal muscles in dogs with acute thoracolumbar intervertebral disk extrusion. The aim of this retrospective study was to describe MRI features of paraspinal muscle signal alteration in dogs with acute thoracolumbar intervertebral disk extrusion and to investigate an association of the signal alterations with neurological grade, type and location of intervertebral disk extrusion, degree of spinal cord compression, and presence of epidural hemorrhage. Medical records of dogs undergoing MRI because of thoracolumbar intervertebral disk extrusion between August 2014 and June 2016 were reviewed. MRI was evaluated for SI changes within the paravertebral musculature, their location, extension, affected muscles, contrast enhancement, and signal void in T2* sequences. Intervertebral disk herniation was categorized as acute non-compressive nucleus pulposus extrusion (ANNPE) or compressive intervertebral disk disease. In five patients, muscle biopsies of areas with signal intensity changes were taken during surgery. In total, 103 dogs were enrolled in the study. Paraspinal muscle signal alterations were visible in 37 dogs (36%) affecting the epaxial musculature (*n* = 17), hypaxial musculature (*n* = 12), or both (*n* = 8). All signal alterations were hyperintense on T2-weighted images and iso- or hypointense in T1-weighted images. Signal void in T2* was not observed in any dog. Postcontrast sequences were available in 30 of the 37 dogs and showed enhancement in 45%. There was neither an association with degree of compression nor epidural hemorrhage. Intervertebral disk extrusion caudal to L1 and a higher neurological grade was associated with the presence of muscle changes. Histopathology revealed mild to moderate acute muscle fiber degeneration with edema and necrosis in three of five samples. The MRI, as well as the muscle samples, show rather unspecific changes. The underlying pathomechanism might be related to ischemia or muscle spasm, but also denervation edema may explain the signal alteration.

## Introduction

Magnetic resonance imaging (MRI) signal intensity of normal skeletal muscle is generally slightly higher than that of water and much lower than fat on T1-weighted (T1W) images and much lower than both fat and water on T2-weighted (T2W) images (1). Alterations of signal intensity of skeletal muscles due to pathologic conditions is easily identified on inversion-recovery and fat-suppressed T2W images (1, 2). The potential causes are diverse, but usually the abnormal signal intensity identified on MRI falls into one of three recognizable patterns: edematous lesions, mass lesions, or fatty infiltration (1). All three patterns have been identified within the paraspinal muscles of dogs on MRI. Edematous lesions of the paraspinal muscles have been observed in immune-mediated polymyositis of the sublumbar muscles (3), associated with meningoencephalitis of unknown origin in the cervical musculature, or due to paraspinal infection in the ventral cervical and lumbar musculature (4). In cases of paraspinal infection with abscess formation, a mass effect caused by cavitary lesions may be identified on MRI (4). Neoplastic lesions result in mass lesions within the musculature either originating from the vertebral column or the paraspinal musculature itself. A third pattern consists of fatty infiltration of paraspinal muscles and has been observed in chondrodystrophic and non-chondrodystrophic dogs, probably driven by a combination of chronicity and severity of spinal cord pathology (5).

In dogs with acute intervertebral disk herniation, we previously observed signal alterations with an edematous pattern in the paravertebral muscles. To the best of our knowledge, this finding has not been reported in the current literature. Therefore, the first aim of this retrospective study was to assess the prevalence of signal alterations within the paravertebral muscles in dogs with acute thoracolumbar intervertebral disk extrusion. Second, to describe their MRI features and investigate possible associations between the intervertebral disk herniation and neurological findings. Furthermore, we performed a histological examination of the musculature with signal alterations in five dogs to investigate the underlying pathological features.

## Materials and Methods

### Patients

Medical records between August 2014 and June 2016 were reviewed and scrutinized for dogs undergoing MRI of the thoracolumbar spine as a result of acute intervertebral disk disease. Dogs were included in the study if they were presented for MRI within 7 days of the onset of clinical signs. Further inclusion criteria were a neurologic examination and that the MRI examination protocol included a fat-suppressed T2W sequence. Dogs were excluded when the medical history suggested changes in the spinal musculature may have occurred. This could be a consequence of a car accident, a fall from a great height, previous spinal surgery within a period of 1 year or previous injections in the paraspinal region. MRI examination was performed in a 1.0-T open permanent magnet (Philips HFO Panorama, Philips Medical Systems, PC Best, Netherlands).

Duration of clinical symptoms and neurological grade was noted based on clinical records. The neurological grade was classified based on neurologic examination as grade 1 (pain only), grade 2 (ambulatory paraparesis), grade 3 (non-ambulatory paraparesis), grade 4 (paraplegia with no loss of deep pain sensation), or grade 5 (paraplegia with loss of deep pain sensation) (6).

### Image Analysis

Images were reviewed by a board-certified radiologist (DSG) for the presence of signal alterations within the paravertebral musculature in fat-suppressed T2W sequences, either Short-TI Inversion Recovery sequence or Spectral Presaturation with Inversion Recovery sequence. If muscle signal alterations were present, their anatomical localization was noted based on the affected muscle, the site along the vertebral column, side, laterality (uni- or bilateral), length, and signal intensity in the different sequences as well as contrast enhancement in postcontrast sequences, if available, were assessed.

Furthermore, the location and type of intervertebral disk herniation [compressive versus non-compressive intervertebral disk extrusion] was noted. Acute noncompressive nucleus pulposus extrusion (ANNPE) represents an acute extrusion of normal, non-degenerate, nucleus pulposus material, causing minimal to no spinal cord compression. The diagnosis of ANNPE have been made based on established MRI criteria consisting of (i) a focal area of intramedullary spinal cord hyperintensity on T2W images that overlies an intervertebral disk space, (ii) a reduction in volume of the T2W hyperintense nucleus pulposus signal, (iii) mild narrowing of the associated disk space, and (iv) extradural material or signal intensity change with minimal or no spinal cord compression at this level (7). The location of compressive material within the vertebral canal and degree of spinal cord compression was described, as well as establishing the presence or absence of epidural hemorrhage within the vertebral canal. Similar to a previously described grading scheme, the degree of spinal cord compression was subjectively assessed as grade 1 (less than 30% impingement), grade 2 (30–40%), grade 3 (40–50%), and grade 4 (>50%) (8).

Muscle signal alterations were compared to the location of intervertebral disk extrusion. They were described as being cranial, at the same level or caudal and compared to the location of compressive material in terms of laterality (ipsilateral, contralateral or bilateral).

To investigate possible influencing factors on the prevalence of muscle signal alterations, the association between dogs with and without muscle signal alterations and type of disk extrusion, location of intervertebral disk extrusion, degree of compression, presence of epidural hemorrhage, duration of clinical symptoms in days, and neurological grade was calculated with a Chi-Square test. For factors with low cell frequency (<5) and binary outcome (type of disk extrusion) the Fisher’s exact test was performed. For low cell frequency (<5) and non-binary outcome, the number of categories was reduced to increase the counts in the remaining. Grouping was performed for the parameter “location of disk extrusion” according to the presence or absence of ribs into T3–T13 and L1–L7. The parameter “neurological grade” was grouped into ambulatory dogs (grades 1 and 2) versus non-ambulatory dogs (grade 3, 4, and 5).

Factors which were significantly associated (*P* < 0.05) with the presence of muscle signal alterations in the univariable analysis were offered to a multivariable logistic regression model. Variables were selected by stepwise backward selection, until only significant variables and confounders (variables changing the effect of other variables by >20%) remained in the model. Model fit was assessed by deviance statistics, chi-square goodness of fit test, and visual assessment of residuals. Statistical analysis was performed in NCSS 10 Statistical Software (2015) (NCSS, LLC. Kaysville, Utah). Differences were considered statistically significant if *P* < 0.05.

### Histological Examination

In five patients, biopsies of muscle parts exhibiting signal alterations were taken during decompressive surgery following MRI. Samples were fixed in 10% formalin, processed routinely, stained with hematoxylin and eosin and assessed by a board-certified pathologist.

## Results

### Dogs

In total, 103 dogs (49 females and 54 males) met the inclusion criteria. Mean age was 6.3 years (range: 1–13 years). Affected breeds were French Bulldog (*n* = 28), Dachshund (*n* = 15), Mixed-breed dog (*n* = 12), Shih Tzu (*n* = 8), Labrador Retriever (*n* = 4), Bolonka Swetna (*n* = 3), Coton de Tuléar (*n* = 2), Cocker Spaniel (*n* = 2), Yorkshire Terrier (*n* = 2), Poodle (*n* = 2), Schnauzer (*n* = 2), Basset Hound (*n* = 2), and one of each of the following American Staffordshire Terrier, Appenzeller Sennenhund, Beagle, Border Collie, Border Terrier, Cane Corso Italiano, Chihuahua, Chinese Crested Dog, German Shepherd Dog, Entlebucher Mountain Dog, Jack Russell Terrier, Lhasa Apso, Maltese Dog, Pug, Papillon, Pekinese, Pinscher, Siberian Husky, Tibetan Spaniel, Tibetan Terrier, and West Highland White Terrier.

### Muscle Signal Alterations

**Figure 1:**
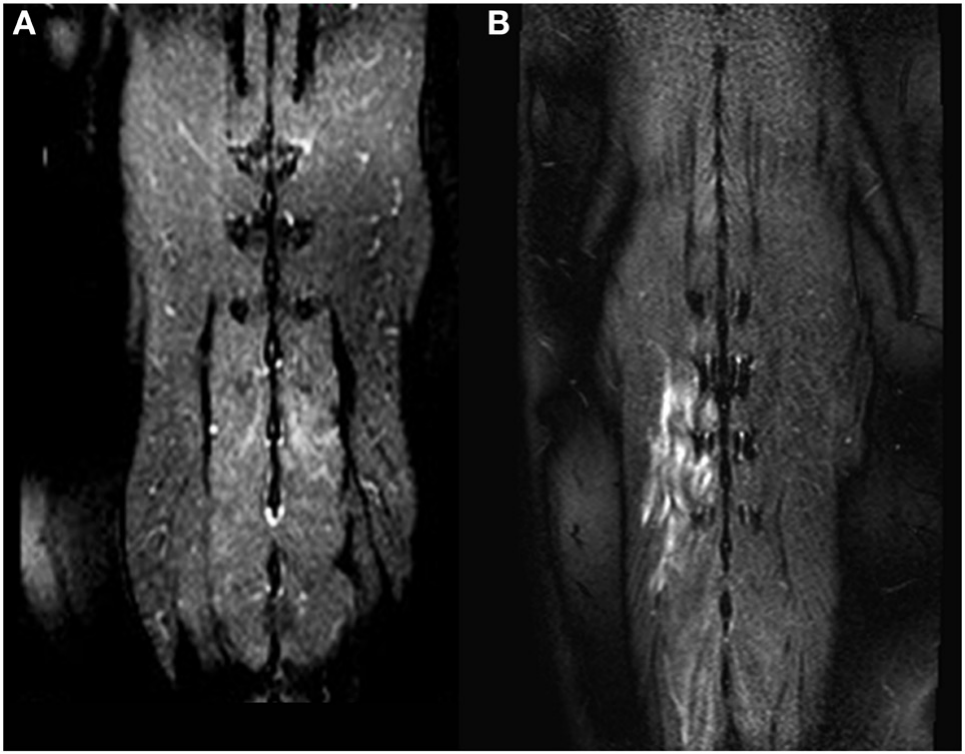
Dorsal T2-weighted fat-suppressed images of two different dogs **(A,B)**. In **(A)**, only a mild signal alteration is visible in the *Mm. multifidi* of a French Bulldog with acute intervertebral disk herniation at the level L2/L3. More extensive signal alterations are visible in the *Mm. multifidi* of a Basset hound **(B)** with intervertebral disk extrusion at the level T12/T13. Both dogs had a neurologic grade of IV.

**Figure 2:**
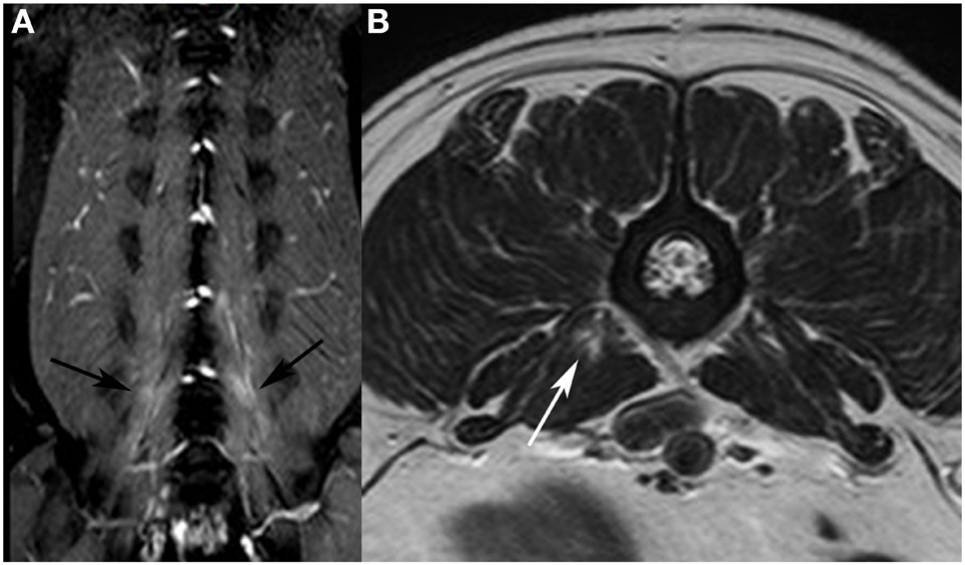
Dorsal T2-weighted (T2W) fat-suppressed image of a French bulldog presented with intervertebral disk extrusion at the level L5/L6. **(A)** bilateral signal alterations are visible in the hypaxial muscles (black arrows). On the T2W fast spin echo transverse image **(B)**, only a focal area of increased signal intensity is visible in the *M. psoas major* on the right side (white arrow).

Signal alterations within the paraspinal muscles were noted in 37 of 103 dogs (36%). The signal intensity was hyperintense on T2W images in 29 dogs, with changes only visible on fat-suppressed sequences in eight dogs (22%) (Figures 1 and 2). T1W images were available in 35 dogs (95%) with hypointense signal alterations observed in six dogs (17%). For the remaining 29 dogs (83%), lesions appeared isointense on T1W images.

Contrast medium was administered in 30/37 dogs with signal alterations. Enhancement was observed on either a T1W sequence or WATS (selective water excitation pulse) in 13 dogs (43%), and no enhancement was seen in 17 dogs (57%). T2* sequences were available in all dogs, a signal void in T2* was not detected in any dog.

### Anatomical Localization

In 18 dogs (49%), unilateral signal alterations were observed (nine dogs right and nine dogs left); bilateral alterations were seen in 19 dogs (51%). The observed signal alterations were seen in one muscle (*n* = 13; 35%) or in multiple paraspinal muscles (*n* = 24; 65%). Two muscles were affected in 15 dogs (41%), three muscles in four dogs (11%), four muscles in four dogs (11%) and six muscles in one dog (3%). The most commonly affected muscles were *Mm. multifidi* (*n* = 15), *M. longissimus* (*n* = 14), *M. psoas major* (*n* = 11), *M. psoas minor* (*n* = 11) and *M. iliocostalis* (*n* = 2) (Table 1). Signal alterations were observed in the epaxial muscles in 17 dogs (46%) (Figure 1), in the hypaxial muscles in 12 dogs (32%) (Figure 2), and in both muscle groups in 8 dogs (21%).

**Table 1:** List of all dogs with muscle signal alteration with the duration of their clinical signs, neurologic grade, degree of compression, location of disk extrusion, level of muscle signal alteration in the vertebral column, number of adjacent vertebral bodies, affected muscles, and laterality.

Dogs with muscle signal alteration	Duration of clinical signs in days	Neurologic grade	Degree of compression	Location of disk extrusion	Level of muscle signal alteration	Number of adjacent vertebral bodies	*M. multifidus*	*M. longissimus*	*M. iliocostalis*	*M. psoas major*	*M. psoas minor*	Laterality
1	1	4	ANNPE	L1/L2	L1	1	x					Unilateral
2	1	5	3	L2/L3	T13–L3	4					x	Bilateral
3	1	4	4	T11/T12	T12–L1	3		x			x	Unilateral
4	1	4	3	L1/L2	L2	1					x	Unilateral
5	2	5	4	L4/L5	L6–L7	2					x	Bilateral
6	1	3	1	L3/L4	L2–L3	2		x				Unilateral
7	1	4	ANNPE	L2/L3	L4	1	x					Unilateral
8	1	1	3	L1/L2	L1 - L3	3	x					Unilateral
9	1	3	3	L5/L6	L5–L6	2		x			x	Bilateral
10	1	3	3	L3/L4	L3- L4	2		x		x	x	Unilateral
11	1	5	4	T12/T13	L2–L3	2				x		Bilateral
12	1	4	4	T12/T13	T12–T13	2	x					Unilateral
13	1	4	2	T12/T13	IVD L1/L2	1					x	Unilateral
14	1	4	2	L3/L4	L3–L5	3				x	x	Bilateral
15	1	5	1	T12/13	T11–L2	5	x	x				Bilateral
16	4	2	3	T12/T13	T12–T13	2	x					Bilateral
17	5	4	2	L1/L2	L3–L4	2	x	x			x	Bilateral
18	7	3	4	L4/L5	L4–L6	3	x	x				Bilateral
19	7	4	4	T12/T13	L1–L2	2	x					Bilateral
20	1	3	2	L3/L4	IVD L3/L4	1		x		x		Bilateral
21	4	4	4	L2/L3	L1–L3	3				x		Bilateral
22	7	4	4	L2/L3	L2–L3	2	x	x		x		Unilateral
23	1	4	3	L5/L6	IVD L5/L6	1				x		Unilateral
24	1	5	3	T11/T12	L1–L3	3	x					Bilateral
25	1	4	3	L1/L2	L1–L3	3				x		Bilateral
26	1	4	2	L1/L2	T12–L2	4	x					Unilateral
27	1	4	3	L1/L2	IVD L1/L2	1			x		x	Unilateral
28	3	4	2	L4/L5	IVD L2/L3	1					x	Unilateral
29	2	4	2	L2/L3	L2-4	3	x	x				Bilateral
30	1	5	1	L3/L4	L2 -L4	3		x	x			Unilateral
31	1	5	3	L2/L3	L2–L3	2		x		x		Bilateral
32	1	4	3	T12/T13	L1–L3	3	x					Bilateral
33	2	3	1	L2/L3	L2–L3	2	x					Unilateral
34	1	3	4	T13/L1	L1	1		x				Unilateral
35	2	3	2	L2/L3	L5–L7	3				x		Bilateral
36	1	3	1	L3/L4	L2–L4	3				x		Bilateral
37	1	4	1	T13/L1	L1–L3	3		x				Unilateral

The most cranially observed signal alteration was at the level of the eleventh thoracic vertebra, and the most caudal located changes were observed at the level of the seventh lumbar vertebra. The median length of signal alterations was two vertebral segments, with a range from one to five (Table 1).

### Association with Intervertebral Disk Extrusion’s Features

**Figure 3:**
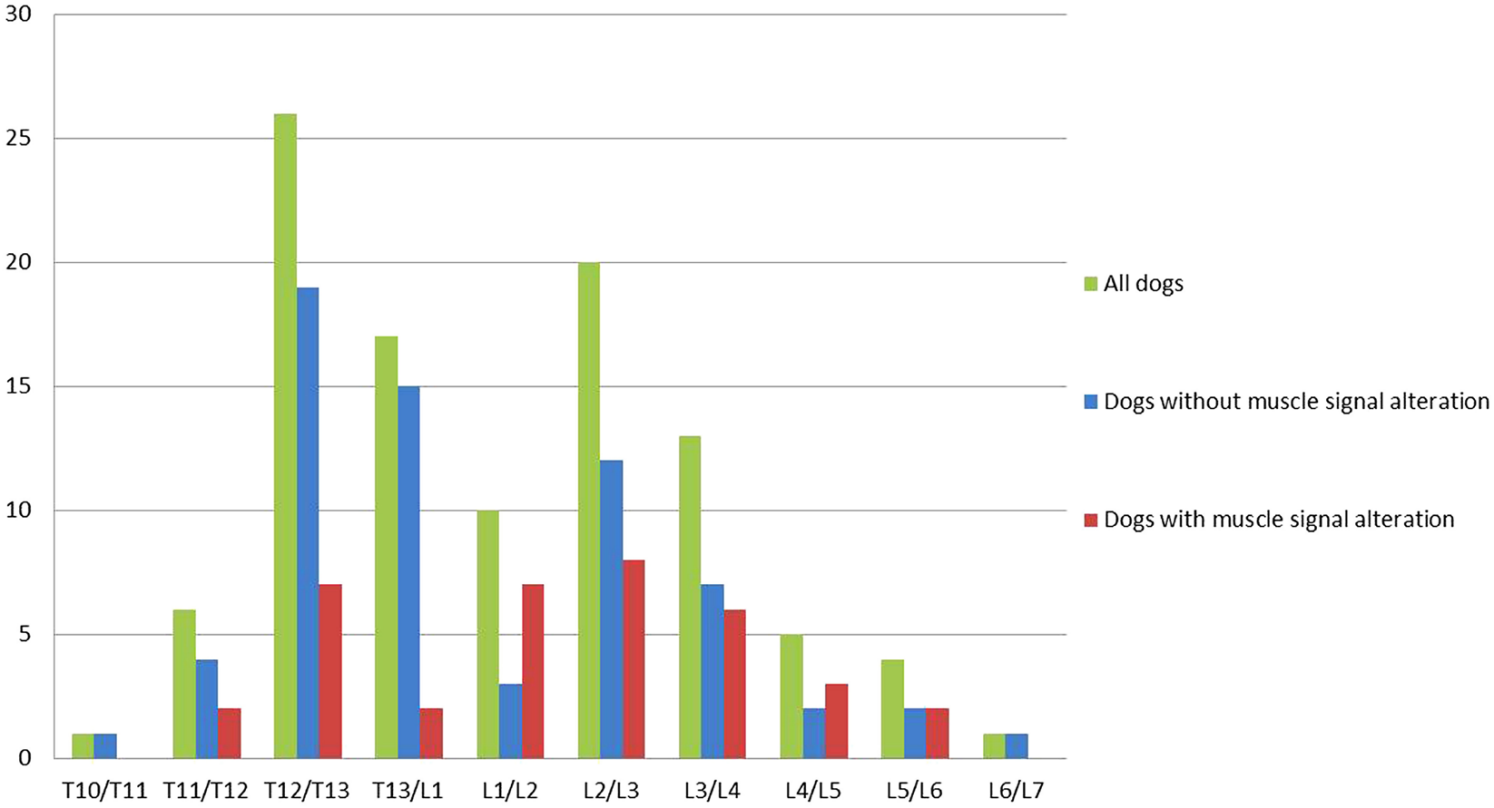
Graph illustrating the location of the intervertebral disk extrusion of all dogs, dogs without and with muscle signal alteration. Dogs with a disk extrusion caudal to L1 had a higher risk for muscle signal changes (OR = 2.8, *P* = 0.03) than dogs with a disk extrusion cranial to L1.

Intervertebral disk extrusion was most often located at T12/T13 (*n* = 26; 25%), followed by L2/3 (*n* = 17; 17%) as the second most common site. In dogs with muscle signal alterations most intervertebral disk extrusions were present at L2/L3 (*n* = 8; 22%), followed by L1/L2 and T12/T13 (each *n* = 7; 19%) (Figure 3). The proportion of dogs with muscle signal changes was significantly higher in dogs with an intervertebral disk extrusion between L1 and L7 compared to T3–T13 (Chi-square test, *P* = 0.004).

Location of muscle signal alterations in relation to location of intervertebral disk extrusion is visible in Table 1. Muscle signal alterations occurred most commonly at the same level as the intervertebral disk extrusion (*n* = 25; 68%) extending to a different degree cranial and/or caudal (Table 1). In two dogs (5%), the muscle signal alterations were located cranial to the affected intervertebral disk without involving the disk level, and in a further 10 dogs (27%), the signal alterations were located caudal to the affected intervertebral disk space. The muscle signal alterations were seen along one to four adjacent vertebrae with a median of two (Table 1).

Compressive extrusions were present in 91 dogs (88%), whereas 12 dogs (12%) had an ANNPE without compression of the spinal cord. Only one dog demonstrated ANNPE signal alterations within the paraspinal muscles and type of disk extrusion was not associated with muscle signal alterations (Fisher’s exact test *P* = 0.20372).

Dogs presented with varying degrees of spinal cord compression; less than 30% impingement (*n* = 21; 20%), between 30 and 40% impingement (*n* = 26; 25%), between 40 and 50% (*n* = 29; 28%), or more than 50% (*n* = 27; 26%). The degree of compression was not associated with the presence of muscle signal alterations. In 13 dogs, muscle signal alterations were ipsilateral to the side of compression and in a single dog on the contralateral side. Despite unilateral compression, the muscle changes were observed bilaterally in 18 dogs. Ventral compression resulted in either unilateral or bilateral muscle signal changes.

Epidural hemorrhage was identified in 44 dogs (43%). Epidural hemorrhage was not associated with muscle signal alterations (Chi-square test, *P* = 0.738).

### Association with Neurological Grade

In dogs with muscle signal alterations, the median neurological grade was 4, compared to a median of 3 in dogs without signal changes. The proportion of dogs with muscle signal alterations was higher in non-ambulatory dogs (neurological grades 3, 4, and 5) compared to ambulatory dogs (neurological grade 1 and 2) (Chi-square test *P* = 0.0017).

For both, dogs with an extrusion cranial to L1 and caudal to L1, the median neurological grade was 4.

Most dogs (*n* = 60; 59%) were presented within the first 24 h after the onset of clinical symptoms, the remainder within 48 h (*n* = 18; 18%), 3 days (*n* = 6; 6%), 4 days (*n* = 6; 6%), 5 days (*n* = 4; 3%), 6 days (*n* = 0; 0%), or 7 days (*n* = 9; 9%). Duration of clinical symptoms was not associated with the presence of muscle signal changes.

The two factors significantly associated with the presence of muscle signal changes in the univariable analysis were also significant in the multivariable logistic regression model. Dogs with a disk extrusion caudal to L1 had a higher risk for muscle signal changes (OR = 2.8, 95%CI = 1.1–7.0, *P* = 0.03) than dogs with a disk extrusion cranial to L1. Neurological grades 1 and 2 were grouped, and used as the reference group. Grades 3, 4, and 5 were compared to the reference group, respectively. Dogs with a neurological grade of 3, 4, or 5 had a higher risk for muscle signal changes compared to dogs with a grade of 1 or 2 (*P* = 0.002). The Odds ratios for neurological grades 3, 4, and 5 were 7.3 (95%CI = 1.3–39.1), 12.1 (95% CI = 2.4–60.2) and 10.5 (95%CI = 1.7–62.7), respectively.

### Histopathology

Histopathological examination revealed a focal to focally extensive, mild to moderate acute muscle fiber degeneration and necrosis in three samples, characterized by fragmentation of the sarcoplasm. The basal lamina and the endomysium were intact in most cases. Between the degenerated fibers, a mild to moderate accumulation of fibrin, neutrophilic granulocytes and edema was present. In addition, there was extensive hemorrhage in one sample and two muscle samples showed mild edema.

## Discussion

Paraspinal muscle signal alteration was observed in 36% of dogs with acute intervertebral disk extrusion and is, therefore, considered a common finding on MRI examination. The MRI pattern of these changes corresponds to a muscle edema. This pattern of T2-hyperintense and T1-iso- to hypointense signal abnormality is an unspecific alteration and the potential underlying causes are diverse. This finding has not been previously described in dogs or humans in association with acute intervertebral disk extrusion. The underlying cause for the signal changes is unknown; therefore muscle samples from the affected musculature were taken prospectively in five dogs to investigate the underlying pathomechanism. The results of the histopathologic examination were rather unspecific with changes including edema and focal muscle necrosis. An explanation for these changes may be trauma, ischemia or denervation, but the exact cause could not be determined by histopathology.

Penetration of extruded intervertebral disk material into the musculature may directly induce trauma and the associated signal alterations. However, this seems unlikely since the signal alterations were often observed bilaterally or even contralaterally to the extrusion. Furthermore, lesions were seen caudally or cranially to the extruded intervertebral disk without being in contact to the intervertebral disk space. Muscle signal alterations were observed in the majority of dogs within the epaxial muscles, dorsal to the level of the transverse processes of the vertebrae, but in 12 dogs, signal alterations were observed only in the hypaxial muscles, ventral to the transverse processes. These observations suggest that penetration of extruded intervertebral disk material in to the musculature is unlikely to be responsible for the signal alterations.

Both epaxial and hypaxial muscle groups are innervated by the spinal nerves from their dorsal or ventral branches, respectively (9). Damage to these nerves can lead to denervation edema within the muscle. Muscle denervation is a well-known pathologic condition in humans. The muscles of the foot, upper limb or shoulder are most commonly affected, but can be observed in any skeletal muscle (10). Causes may include trauma or entrapment of peripheral nerves, but spinal cord pathology can also result in nerve injury. The least severe form of nerve injury is neurapraxia (10). In contrast to neurotmesis and axonotmesis, this damage does not lead to structural discontinuity of axons, but instead a conduction block within the nerve causes muscle denervation (10). Denervation edema as a consequence of acute intervertebral disk extrusion has not been described in humans. In humans, where an intervertebral disk herniation at the level L3/4 or further caudal does not result in spinal cord compression, an intervertebral disk extrusion in dogs can cause spinal cord compression even at the level L6/L7 (11). Since the vertebral canal in dogs is much smaller compared to humans, intervertebral disk extrusion often results in marked compression or contusion with damage to the neurons located in the gray matter (11). We observed an association of muscle signal alterations with intervertebral disk extrusion caudal to L1, where the amount of gray matter is increasing along the lumbar spine. An association of muscle signal alterations with more severe spinal cord injury is supported by the fact that a significantly higher number of dogs with muscle signal alterations were presented with a higher neurological grade. Neurological grade or more precise, loss of deep pain perception, is a prognostic factor for neurologic recovery. At this time, it remains unclear if muscle signal alterations may be able to provide information about the neurologic recovery of the patients.

If neuronal or axonal damage occurs without inducing Wallerian degeneration of the axons, the changes in the axons are considered to be reversible (10). Otherwise, the chronic denervation results in atrophy of the muscles and fatty degeneration. A reduced cross sectional diameter and also fatty infiltration of the paraspinal musculature, especially the multifidi muscle group, has been described in humans with lower back pain as well as in experimental animal studies with induced intervertebral disk degeneration or extrusion (10, 12–17). Fatty infiltration within the paraspinal musculature has also been observed in dogs (5, 18), but no association between the degree of intervertebral disk degeneration and fatty infiltration was found and so far, the relationship of intervertebral disk disease and fatty infiltration is unclear (5). It is hypothesized that fatty infiltration in dogs is driven rather by a combination of chronicity and severity of the general spinal pathology (5). To further investigate if muscle signal alterations with an edematous pattern are reversible and if they result in degeneration with fatty infiltration, serial MRI examinations are necessary. Interesting questions to address in the future are the relationship of muscle signal alteration with pain, as well as the prevalence of muscle signal alterations in a larger cohort of dogs with ANNPE. Only one dog from all with signal alterations in the musculature was diagnosed as ANNPE based on MRI. This suggests that non-compressive spinal cord damage results less often in muscle signal alterations. This phenomenon may be explained by less severe damage of the gray matter. However, the overall lower number of ANNPE animals compared to those with compressive intervertebral disk disease does not allow drawing this conclusion.

The muscle signal alterations were best identified in fat-suppressed T2W sequences, which are used as part of our routine protocol in dogs with acute back pain to identify changes in soft tissues (2). The sequences enable changes to be identified in a high proportion of animals, including more than one third of the dogs (36%). In some of the dogs, the lesions showed contrast enhancement, but contrast enhancement of skeletal muscles did not seem to facilitate or further characterize the lesions. This is in contrast to reported cases in humans (10). Identification of paraspinal muscle signal alterations may be helpful in clinical MRI to differentiate intervertebral disk disease from fibrocartilaginous embolism or acute versus chronic intervertebral disk herniation, but further studies are necessary to answer these questions.

In conclusion, we observed muscle signal alterations within the paraspinal muscles in more than one third of dogs with acute thoracolumbar intervertebral disk disease. The changes occur more frequently in intervertebral disk extrusions caudal to L1 and in dogs with a higher neurological grade. The rather unspecific MRI pattern of the edematous lesions as well as the histological findings obtained from five dogs hinders clarification of the underlying pathomechanism, but denervation edema may explain the signal alteration observed in dogs with acute intervertebral disk extrusion.

## Consent

Consent procedure is not applicable, as this article is a retrospective study of clinical cases.

## Ethics Statement

This study was carried out in accordance with the ethic recommendations of the veterinary Office, Kanton Bern, Switzerland.

## Author Contributions

Conception and design: DS-G and PT. Acquisition of data: PT, DH, MW, CG, FF, and DS-G. Analysis and interpretation of data: PT, DS-G, CG, MW. Drafting the Article: PT and DS-G. Revising article for intellectual content: DS-G, PT, and MW. Final approval of the completed article: PT, CG, MW, FF, DH, and DS-G.

## Conflict of Interest Statement

The authors declare that the research was conducted in the absence of any commercial or financial relationships that could be construed as a potential conflict of interest.
